# From CySkin to ProxySKIN: Design, Implementation and Testing of a Multi-Modal Robotic Skin for Human–Robot Interaction

**DOI:** 10.3390/s24041334

**Published:** 2024-02-19

**Authors:** Francesco Giovinazzo, Francesco Grella, Marco Sartore, Manuela Adami, Riccardo Galletti, Giorgio Cannata

**Affiliations:** 1 Department of Informatics, Bioengineering, Robotics and Systems Engineering (DIBRIS), Università di Genova, Via all’Opera Pia 13, 16145 Genova, Italy; giorgio.cannata@unige.it; 2 ElbaTech Srl, Via Roma 10, 57030 Marciana, Italy; sartore@elbatech.com (M.S.); adami@elbatech.com (M.A.); galletti@elbatech.com (R.G.)

**Keywords:** sensor arrays and networks, robotic skin, tactile sensors, proximity sensors

## Abstract

The Industry 5.0 paradigm has a human-centered vision of the industrial scenario and foresees a close collaboration between humans and robots. Industrial manufacturing environments must be easily adaptable to different task requirements, possibly taking into account the ergonomics and production line flexibility. Therefore, external sensing infrastructures such as cameras and motion capture systems may not be sufficient or suitable as they limit the shop floor reconfigurability and increase setup costs. In this paper, we present the technological advancements leading to the realization of ProxySKIN, a skin-like sensory system based on networks of distributed proximity sensors and tactile sensors. This technology is designed to cover large areas of the robot body and to provide a comprehensive perception of the surrounding space. ProxySKIN architecture is built on top of CySkin, a flexible artificial skin conceived to provide robots with the sense of touch, and arrays of Time-of-Flight (ToF) sensors. We provide a characterization of the arrays of proximity sensors and we motivate the design choices that lead to ProxySKIN, analyzing the effects of light interference on a ToF, due to the activity of other sensing devices. The obtained results show that a large number of proximity sensors can be embedded in our distributed sensing architecture and incorporated onto the body of a robotic platform, opening new scenarios for complex applications.

## 1. Introduction

Safety, productivity and synergy are the key elements of a successful human–robot collaboration (HRC). In traditional manufacturing systems, industrial robots lack the necessary sensing infrastructures to achieve effective collaboration with humans and are typically confined to restricted cells for safety reasons. To overcome this limitation, robots are usually equipped with additional sensing devices that improve their understanding of the world, increasing their autonomy and performance while ensuring safety.

External sensing infrastructures, such as cameras and motion capture systems, are commonly used to provide visual feedback to the robotic platform. For instance, studies like [1,2,3,4,5,6] use an external vision-based interface to monitor the collaborative workspace and track the human operator in order to ensure their safety and estimate or predict their intentions. However, these solutions assume a very well-known, perfectly calibrated and structured working environment, which limits the shop floor task reconfigurability and the range of potential applications. In addition, external cameras are affected by light sensitivity, occlusions and blind spots, which can be caused by obstacles or humans standing between the robot and the sensing device, or due to an improper placement of the camera itself. Finally, vision-based systems have limited capabilities in measuring physical interaction phenomena that may occur during human–robot collaborative tasks.

A viable alternative to external vision-based interfaces involves equipping industrial robots with additional sensing devices directly integrated onto the robotic platform. Distributed networks of tactile sensors covering the robot body can provide tactile feedback from a large area and can accurately measure not only where the contact occurs, but also the shape and the force of the contact distribution. The existing literature explores various distributed tactile sensing architectures based on different transduction technologies, including capacitive [7,8], piezoelectric [9,10,11], optical [12,13] and multi-modal [14]. These technologies open new scenarios in physical human–robot interaction (pHRI) and physical contact processing, as the robot can interpret a human’s intention to collaborate or perceive unexpected collisions and react accordingly.

However, the sole use of tactile sensors is not sufficient to prevent unintentional collisions with obstacles surrounding the robot during its motion. Therefore, it is inadequate to ensure safe robot operation in the collaborative workspace, except for very specific applications, as demonstrated in [15].

To increase robots’ autonomy and interoperability in various collaborative scenarios, distributed tactile sensors should be complemented by other sensing devices, leading to a robust, seamless, multi-modal sensing infrastructure. To this end, proximity sensors represent a cost-effective and efficient technology widely used in the literature to bridge the gap left by tactile and vision perception. A sensory skin providing both tactile and proximity information can be integrated onto the industrial manipulator body to enhance its perceptual capabilities. The robot can then leverage the embedded proxi-tactile feedback to probe the surrounding environment, detect the presence of objects, approaching obstacles and humans, and enable a wide range of new possibilities in terms of safety and task implementation. Works described in [16,17,18,19,20,21] showcase different prototypes of distributed proximity sensors and their use in robotic applications to ensure safe human–robot interaction.

Distributed multi-modal sensing architectures provide heterogeneous information about the robot’s peripersonal space, enabling robot systems to operate and interact safely with limited out-of-the-robot infrastructure, and potentially reducing robot setup times and costs, thus increasing the flexibility of the shop floor configuration.

In [14], the authors designed and developed a multi-sensitive hexagonal IC taxel, HEX-O-SKIN, to achieve a multi-modal whole-body-touch sensation in humanoid robots. The robotic skin has a modular structure equipped with MEMS accelerometers, optical reflective sensors for proximity and resistive temperature sensors. The effectiveness of the skin is demonstrated in [22,23,24,25] but the size, rigidity and thickness of the modules do not allow for the placement of the skin on just any robot surface.

In [26], TacSuit is introduced, a multi-modal, modular, large-area skin for collaborative robots that integrates pressure, proximity, acceleration and temperature sensors. This technology is similar to HEX-O-SKIN in dimensions and geometry, but to achieve conformity, scalability and easy installation to the robot surface, the sensing elements are housed in custom-designed 3D printed capsules. TacSuit is integrated onto a humanoid robot and validated with different control strategies in safe human–robot interaction applications.

A multi-modal sensor array capable of contact detection and localization, force sensing, proximity and mapping is presented in [27,28]. The core idea is to replace the passive spacer disks of a continuum robot with multi-modal sensing disk units (SDUs) equipped with the Hall effect and Time-of-Flight (ToF) sensors, ensuring a safe interaction along the entire length of the robot body. However, the proposed solution cannot be adapted for integration into existing robotic platforms. Additionally, the ToFs embedded into the SDUs have limited proximity sensing capability (0–100 mm), restricting the robot’s perception of the surrounding space.

**Figure 1:**
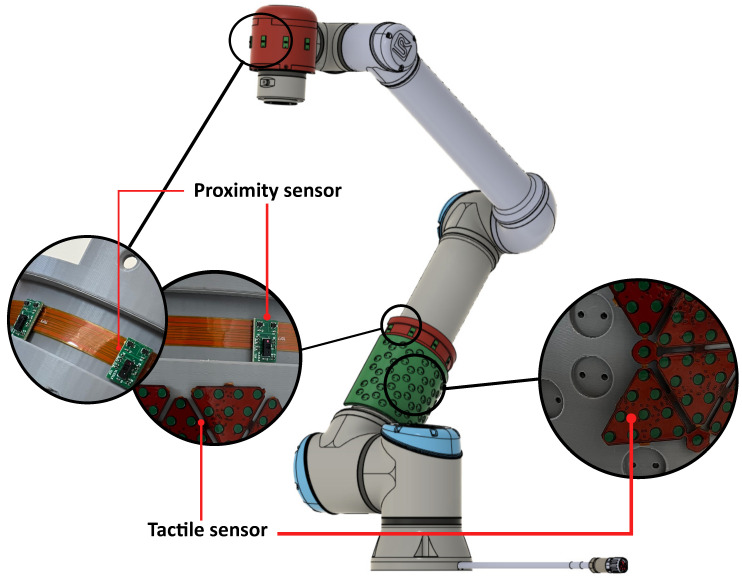
Excerpt of the CAD model of a UR10e robot partially covered with proximity and tactile sensors placed on ad hoc designed covers. The hole pattern on the tactile sensors support is required to lodge electronic components on the rear side of each module.

In this context, we provide first insights into the development of ProxySKIN, a skin-like sensory system based on networks of embedded proximity and tactile sensors. Once integrated into the links of a collaborative robot, as depicted in Figure 1, this technology allows us to control the robot’s actions and interactions safely and autonomously by providing a proxi-tactile perception of the surrounding environment. Compared to the other multi-modal large-area skins, ProxySKIN features a higher spatial resolution of tactile elements: 2.03 pressure sensors$/{\mathrm{cm}}^{2}$, as opposed to 0.78 pressure sensors$/{\mathrm{cm}}^{2}$ of HEX-O-SKIN and 0.19 pressure sensors$/{\mathrm{cm}}^{2}$ of TacSuit. The ToF sensors selected for our architecture have a multi-zone distance measurement capability with up to 64 separate zones, resulting in higher spatial resolution proximity measurements than the technologies previously described. In addition, ProxySKIN is manufactured with flexible printed circuit boards (FPCBs), allowing sensors to conform to smooth curved surfaces. This solution opens new challenges for the development of complex applications and self-standing, autonomous robots with high cognitive capabilities.

In this paper, we present the evolution of our distributed sensing architecture, starting from Cyskin, a well-established tactile technology, and we provide examples of its successful adoption in robotic applications, emphasizing its limitations and the need for a proper multi-modal upgrade. We thoroughly analyze the design of arrays of Time-of-Flight (ToF) sensors embedded in ProxySKIN, based on the sensor specifications and the requirements of robotic applications. We describe the technical details and challenges in the hardware realization of the ToF sensing devices and provide some insights into the communication architecture handling both tactile and proximity sensors. Finally, we evaluate the effects of light interference on ToF measurements, caused by the activity of other proximity sensors.

The rest of the paper is organized as follows: Section 2 and Section 3 present the evolution of our distributed sensing architecture, starting from the CySkin technology up to the CoLLaboratE project [29]. Section 4 focuses on the development of ProxySKIN, particularly on the arrays of proximity sensors embedded in our sensing architecture. Section 5 presents qualitative and quantitative results obtained from the interference characterization of the proximity sensors. Finally, Section 6 contains conclusions and comments about the next possible outcomes of the research.

## 2. CySkin Technology

The first step towards the development of our large-area sensing architecture is taken with CySkin [30], a skin-like input utility for robots in industrial and social interaction applications.

**Figure 2:**
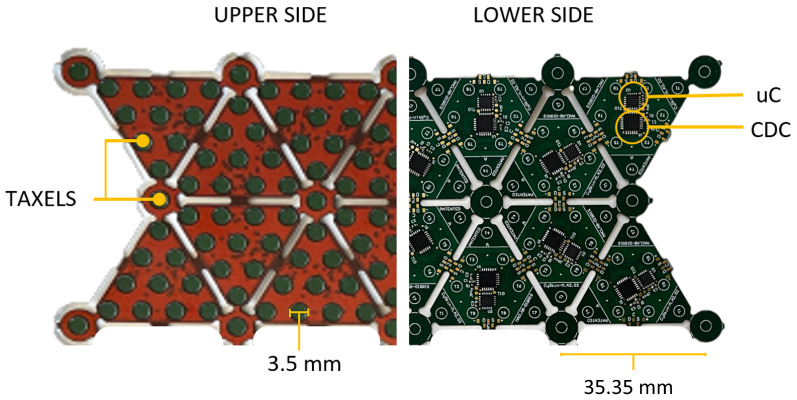
Rear/front view of a CySkin patch with highlighted core components.

**Figure 3:**
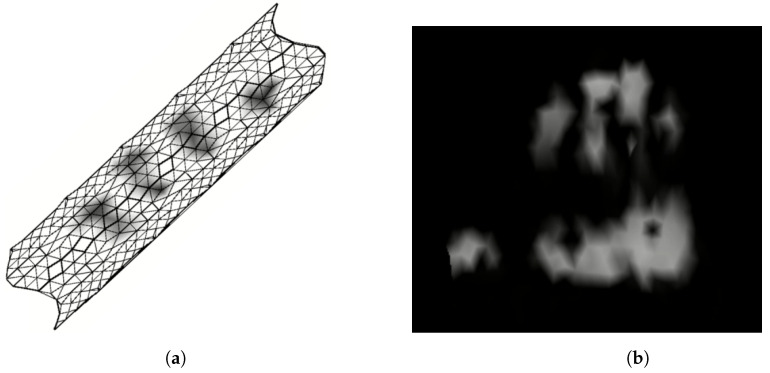
Tactile information representations obtained from CySkin data processing: (**a**) Three-dimensional visualization of the tactile map projected on the mesh of a cylinder covered with CySkin modules. (**b**) Tactile image obtained by a full hand grasp of the cylinder.

To provide robots with the sense of touch, CySkin uses distributed capacitive sensors with the following characteristics: One plate of the capacitor is represented by a circular conductive pad with a diameter of 3.5 mm, etched over a flexible PCB. The second plate of the capacitor consists of conductive lycra connected to the ground and stretched over the PCB. Deformations of the lycra caused by contact pressure generate detectable capacitance variations, according to the capacitive transduction principle, allowing for the estimation of the forces applied to the skin. A capacitance-to-digital converter (CDC) with a 16-bit resolution is used to measure the capacitance of 11 sensors, referred to as *taxels*, arranged in a triangular-shaped module, as shown in Figure 2. The resulting sensing architecture is characterized by a modular, flexible structure that can be adapted and integrated onto curved surfaces and complex geometries. Compared to previous prototypes [31], the addition of an on-board microcontroller embeds local processing, enables the synchronization of all the sensor measurements and, more generally, a better management of network. A group of interconnected modules, called *patch*, communicates with the *Intelligent Hub Board* (IHB), a microcontroller programmed to periodically query measurements from up to 128 triangular elements and send the collected data to a host PC via a CAN bus. This distributed sensing architecture is used to retrieve a dense representation of touch information in the form of tactile maps. Tactile maps provide an interpolation of raw pressure data over a 3D mesh representing the object on which the sensors are positioned. An unfolding algorithm is then employed to retrieve a tactile image, a 2D representation of contacts where each pixel value corresponds to the force/pressure measured by the capacitive sensors of the tactile system, as depicted in Figure 3.

The CySkin technology has been extensively used and tested in various domains and applications. Examples include the navigation of a robotic arm in an unknown and unstructured environment, using a tactile feedback control law to safely handle unexpected collisions with static objects in the surrounding area [32], as well as the discrimination of voluntary or unintentional physical human–robot interaction, by classifying and segmenting human hands in contact with the robot body [33].

## 3. CoLLaboratE Project

**Figure 4:**
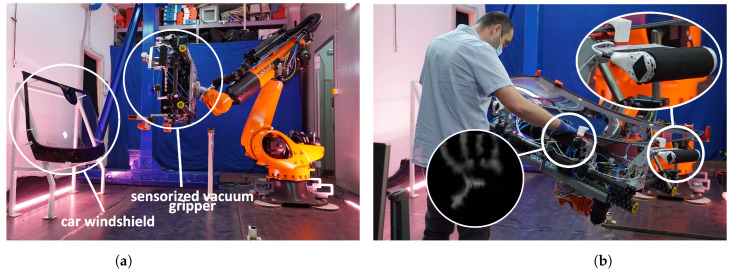
The shared-payload task implemented as use-case scenario for the CoLLaboratE project. (**a**) KUKA KR QUANTEC robot equipped with a vacuum gripper approaching the car windshield to pick it up. (**b**) Collaborative phase: the operator grasps the handles covered with CySkin to effortlessly move the payload. Safety is guaranteed by the detection of a hand shape in the stream of tactile images, which smoothly enables the admittance controller [34].

The CySkin architecture has been employed in the use-case scenario of the EU H2020 Project CoLLaboratE [29], an industry-relevant task focusing on human–robot collaborative assembly in car manufacturing scenarios [34]. In this demonstrator, a heavy-duty industrial manipulator equipped with four handles covered with our artificial skin serves as a weight-lifting system that absorbs the weight of a car windshield, as shown in Figure 4. The human operator, on the other hand, is tasked to inspect the windshield and assemble various components, such as the rear-view mirror and the rain sensor. By grasping one or more sensorized handles, operators can communicate their intention to interact with the robot and adjust the position and orientation of the robot end-effector according to their needs, increasing both the flexibility and ergonomics of the assembly operation.

In this scenario, human contact is used as a novel communication interface and voluntary physical interaction is assumed when the shape of a human hand is classified by a convolutional neural network, as shown in [15,33], which means that the operator is intentionally grasping the sensorized handle to move the robot.

However, relying solely on distributed tactile sensors in the human–robot collaborative task significantly limits the potential applications that could be developed by equipping the industrial manipulator with other types of sensors. For example, in the CoLLaboratE use-case demo, human operators are instructed to press a button before entering the workspace shared with the robot to notify that they are ready to perform their tasks. This operation, besides slowing down the assembly cycle time, does not leverage the cognitive capabilities that the robot might acquire with a more comprehensive and complex sensing architecture. Moreover, it makes the robot dependent on an external signal, thereby reducing its self-sufficiency and autonomy.

## 4. ProxySKIN: Next Generation of CySkin

**Figure 5:**
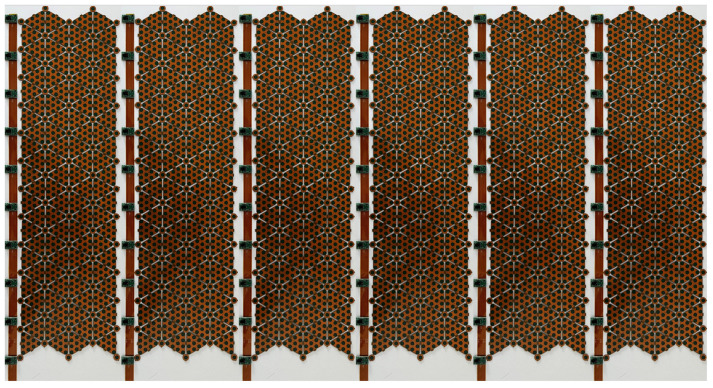
ProxySKIN technology in an integration-ready arrangement.

The realization of ProxySKIN is one of the main goals of the EU Horizon Europe project SestoSenso [35], focused on the development of technologies for next generations of collaborative robots. ProxySKIN consists of a skin-like sensory system based on networks of proximity and tactile sensors, providing the robot with seamless and heterogeneous proxi-tactile feedback about the surrounding space. This technology is built on top of CySkin, which provides tactile information, as well as arrays of the latest generation of Time-of-Flight (ToF) sensors that allow the robot to acquire knowledge about the geometry of the environment and detect obstacles in its vicinity. Patches of distributed tactile sensors are interspersed with arrays of ToFs, as depicted in Figure 5, providing a 360° coverage of the robot’s peripersonal space.

The current version of ProxySKIN is developed on top of a pre-existing technology. To achieve it, we have built two separate networks, each with its own dedicated firmware architecture. This design choice allows us to leverage the capabilities of CySkin without changing its hardware structure. Proximity sensors are then seamlessly integrated into the system at the network level in a plug-and-play fashion. By splitting the two modalities, we could concentrate the development efforts uniquely on the supporting structure of the ToF sensors.

### 4.1. Time-of-Flight Sensors

The ToF sensors selected for our architecture are the VL53L8CX by STMicroelectronics [36]. These proximity sensors feature a multi-zone distance measurement capability with 16 or 64 separate zones at a maximum frame rate of 60 Hz or 15 Hz, respectively, a Field of View of 45° (65° on the diagonal) and a range of up to 400 cm, with enhanced performance under ambient light conditions. Distributed in linear flexible arrays of 10 ToFs, thanks to the multi-zone sensing capability, these sensors can be used to construct a sparse point cloud map of the environment and retrieve useful information for the robot controller, outperforming the results obtained with similar technologies characterized by a single distance measurement, as in [19,27,37].

**Figure 6:**
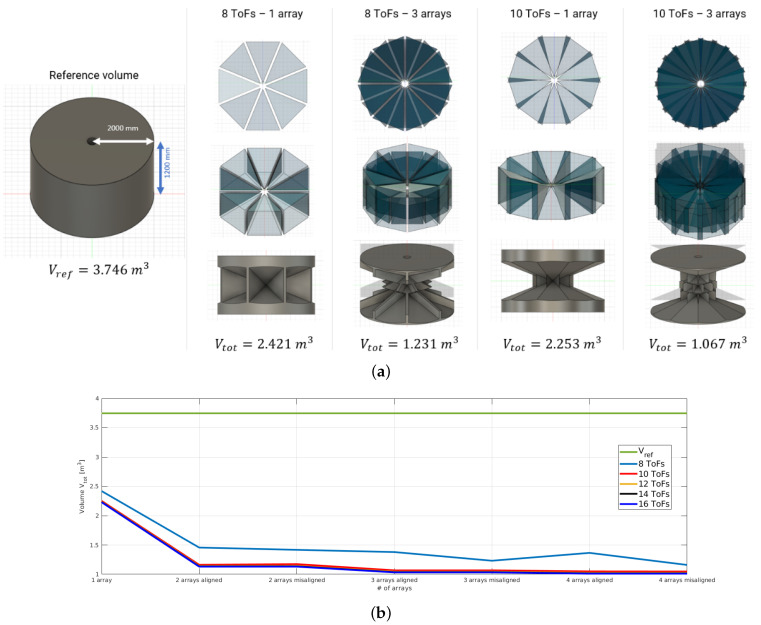
Volumetric analysis of the coverage of circular arrays of proximity sensors. (**a**) Qualitative results showing the reference volume $V_{ref}$, the projection of the Field of View (FoV) of *n* arrays with *m* ToF sensors and the blind volume $V_{tot}$ obtained by subtracting from $V_{ref}$ the projection of the ToF sensors FoV. (**b**) Plot of the blind volume $V_{tot}$ as a function of the number of arrays and the number of ToF sensors per array.

**Figure 7:**
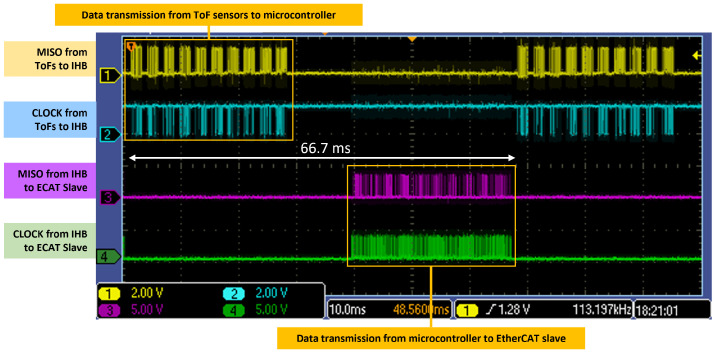
OScilloscope screen capture. Data transmission analysis from an array of 10 ToF sensors to its associated microcontroller (IHB) and from the IHB to the EtherCAT slave device. *Yellow and cyan lines*: SPI signals (MISO and CLOCK) from the array of proximity sensors to the IHB. *Magenta and green lines*: SPI signals (MISO and CLOCK) from the IHB to the EtherCAT slave.

The choice of embedding 10 ToFs into a single array mainly depends on the following sensor characteristics:

- **ToF Field of View** (**FoV**). The FoV of a single proximity sensor is 45°. Therefore, assuming the linear arrays are shaped into a circular pattern, with the sensing elements facing outwards, at least eight ToFs would be necessary to cover the outlying 360°. However, from a volumetric analysis of the ToF coverage, we found that with 10 ToFs per array, the ratio between blind spots and visible spots is significantly reduced. The analysis considers a constant cylindrical volume $V_{ref}=3.746\;m^{3}$, consistent with the space monitored by the proximity sensors on a link of the robot manipulator, and a varying number of arrays (from one to four) and ToFs per array (from 8 to 16). By intersecting and subtracting from the reference volume ($V_{ref}$) the projection of the ToFs FoV, we obtain the blind volume ($V_{tot}$) that is not covered by the proximity fields. The results are reported in Figure 6 and Table 1, showing that three arrays of 10 ToFs represent a good compromise in terms of volume coverage and number of ToFs per array.
- **ToF Power Consumption**. The typical power consumption for a single proximity sensor is about 215 mW, leading to a power consumption of 2150 mW for an array.
- **ToF Data Payload and Transmission Frequency**. The VL53L8CX proximity sensor can provide several data other than the target distance measurement, including the light ambient noise, the variance and the validity of the measured target distance and the estimated reflectance of the target. We consider three fields of the message containing relevant information for robotic applications. In particular, from a single sensor reading in its 64-zone configuration, we extract the target distance, the status and the variance of the measurement, reaching a payload of 320 bytes. With 10 sensors, the full data payload amounts to 3220 bytes, which must be transmitted within 66.7 ms to the microcontroller, assuming to work at the maximum frequency of 15 Hz. Each array of ToF sensors is driven by a microcontroller (the IHB) that sequentially reads the range data through an SPI channel operating at a frequency of 3 MHz. Data are then forwarded to an EtherCAT slave through another SPI channel. This configuration represents a trade-off between system complexity (larger number of ToF sensors for each array) and time lag introduced to transmit the ToF data to be processed by the robot controller. From a temporal analysis of the SPI signals, reported in Figure 7, we can see a significant bandwidth consumption with 10 sensors that does not allow the integration of additional ToFs to the array.

### 4.2. Hardware Design of the Proximity Sensor Arrays

**Figure 8:**
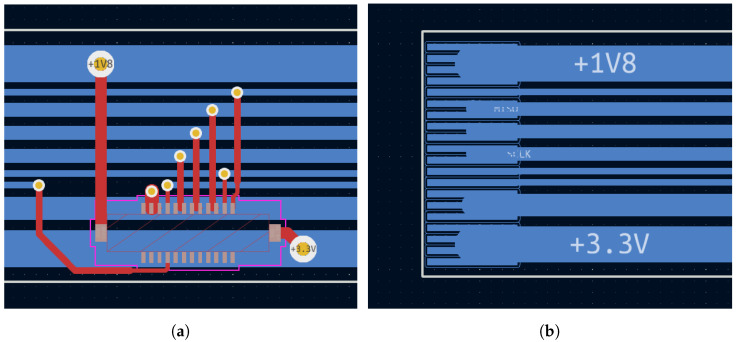
(**a**) The ToF connector on the top layer grabs supplies and signals from the straight bottom tracks; (**b**) the 20-pin end connector groups the little pins together to wider tracks in order to sustain signal integrity and current requirements.

The PCB design of the proximity sensing architecture is based on several constraints, the most demanding of which is certainly space saving. For this reason, particular care is taken to limit the number of parts used, select the smallest component dimensions when possible, optimize the footprint spacing, and finally, split the circuit into blocks suitable to be physically mounted on top of a robot arm. The primary consideration is to position the selected number of ToF sensors equally spaced in a ring-like bendable structure. The best solution is based on a flexible PCB with tiny connectors to accommodate the sensors, granting the possibility to change them in case of need or for testing purposes by a simple click. Thus, the resulting flex PCB measures approximately 50 cm in length, with hosting connectors of 10 proximity sensors spaced at 4.8 cm. The flex PCB is quite long for the clock speeds in use and requires particular care to prevent reflections that could possibly impact the data streams. In addition, two supplies need to be carried along the whole strip: a core 1.8 V and a digital 3.3 V supply for the ToF sensors, once plugged. Combining these two requirements, the Flex PCB was routed, keeping all the tracks perfectly horizontal, serving the 10 ToF connectors in a drop-down fashion as shown in Figure 8a.

The end flex connector in Figure 8b is selected with a number of pins much higher than the number of signals to route, in order to combine multiple pins together, connecting to wider tracks which allow a substantial current flow. Using SPI as the communication interface bus implies that every slave device (in our case, the ToF sensor) owns a dedicated Chip Select signal to share the input–ouput data lines (MISO and MOSI). To limit the overall strip dimensions while using 10 sensors, we implemented a sequential addressing mechanism and generated the *n*th Chip Select signal toggling a D-type flip-flop in the (n−1)th sensor’s board. This solution does not allow a sensor to be randomly addressed at a desired asynchronous time, but only after its predecessor was previously addressed. Then, sequential reading is the only possibility with the described schematics, which is exactly what the present application needs. We instead utilize the big advantage of substantially limiting the wiring budget along the chain, thus supporting our need for little dimensions and secure signaling.

**Figure 9:**
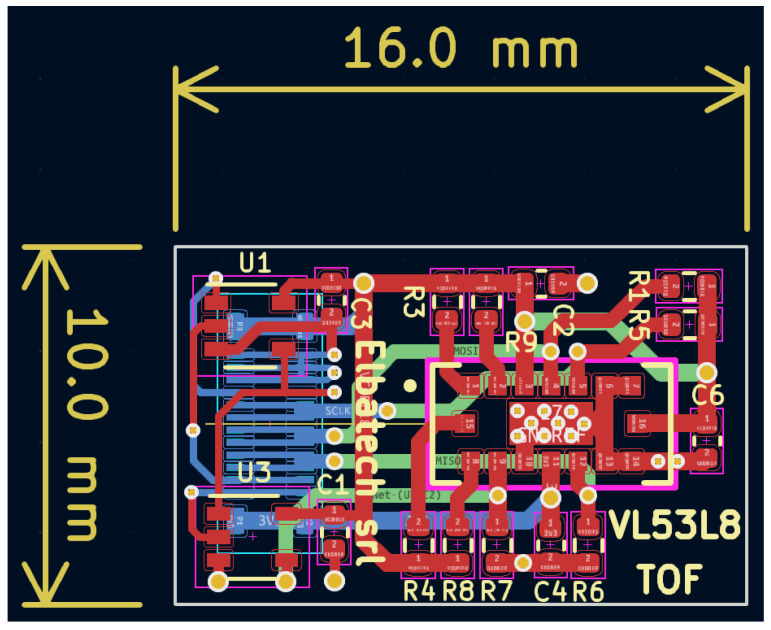
PCB routing of the ToF sensor board. It has a 4-layer stack (ground plane not visible in the figure for clarity). All the SMD parts are placed on the top layer, while only the connector to the flex strip is on the bottom layer to avoid extra height between the PCB and flex that could prevent a proper mating between the male plug and the female socket.

A further consideration worth mentioning is the direct multi-drop connection of the tiny ToF sensors boards at the SPI lines. In fact, the tracks’ linearity along about 50 cm of flex PCB resemble a backplane system where multiple nodes are connected over short distances in a bus topology network, rather than a long and critical transmission line. In this case, the signal integrity can be granted through accurate routing, avoiding the need of multipoint, low-voltage differential signaling (M-LVDS) transceivers (driver and receiver pairs) that would require additional room both on the Flex and on the ToF PCBs. Moreover, placing such transceivers on the Flex near the corresponding sensor connectors would alter and worsen the necessary bending capabilities needed to fix the strips on the robot’s cylindrical arm. Hence, the ToF PCB results are very small, just 10 × 16 mm as shown in Figure 9. To keep good signal quality, a ground plane is spread in one of the inner layers.

### 4.3. Communication Protocols and System Level Description

The main difference between CySkin and ProxySKIN from a communication perspective is that the fist technology uses a CAN bus to transmit the data collected from the tactile sensors to the host PC, while ProxySKIN uses EtherCAT, an Ethernet-based fieldbus suitable for both hard and soft real-time computing requirements in automation technology. The EtherCAT standard, with a data rate of 100 Mbps, is the fastest industrial Ethernet technology and is chosen to unify the data acquisition from tactile and proximity sensors, overcoming the limitations of prior CAN-based solutions.

Patches of proximity and tactile elements are handled by the same microcontroller, the Intelligent Hub Board (IHB) described in [30,38], loaded with a specific firmware depending on the addressed sensing device. The IHB collects data from the sensors through up to four SPI channels, rearranges them into a dedicated buffer, and forwards all the measurements to an associated EtherCAT slave board through another SPI channel. The EtherCAT slave, featuring an Infineon XMC4300 microcontroller, after receiving data from the IHB, dispatches all the information to the host PC, running an EtherCAT master application.

**Figure 10:**
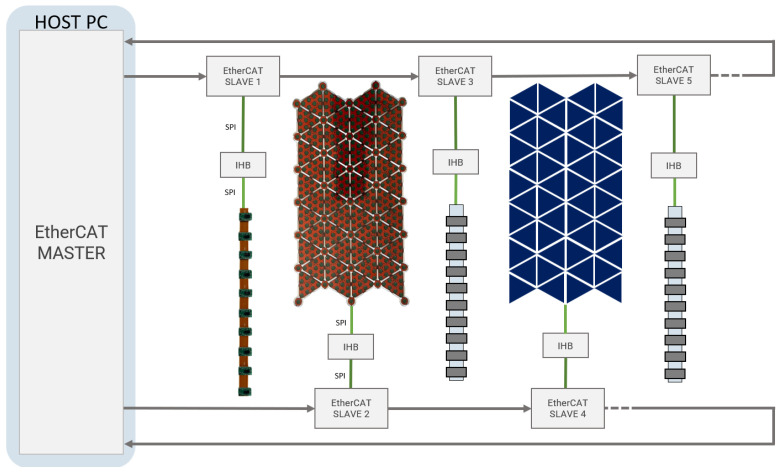
System level integration. Two separated EtherCAT backbones are used for the data acquisition, respectively, for CySkin and arrays of proximity sensors.

All the EtherCAT slaves are interconnected in a daisy chain configuration, resulting in a closed-ring topology that starts and ends with the EtherCAT master device, as depicted in Figure 10. To deal with different acquisition frequencies of proximity and tactile sensors, 15 Hz and 20 Hz, respectively, the current sensing architecture uses two different sensor-specific EtherCAT backbones to transmit data to the EtherCAT master.

The EtherCAT routing is confined inside the robot’s arm and, due to the short distances, it is comparable to a backplane connection from a signal standpoint. For short distances or backplane-oriented EtherCAT applications, communication can be implemented without using isolation transformers and the “big” RJ45 connectors and plugs typical of the Ethernet bus. Instead, low-cost and space-saving interconnections are realized by combining simple ceramic capacitors with physical layer drivers, thus implementing the operation through direct Phy-to-Phy connections. We utilize the DP83848C Single Port 10/100 Mb/s physical layer transceivers for the job.

## 5. Proximity Sensors Characterization

**Figure 11:**
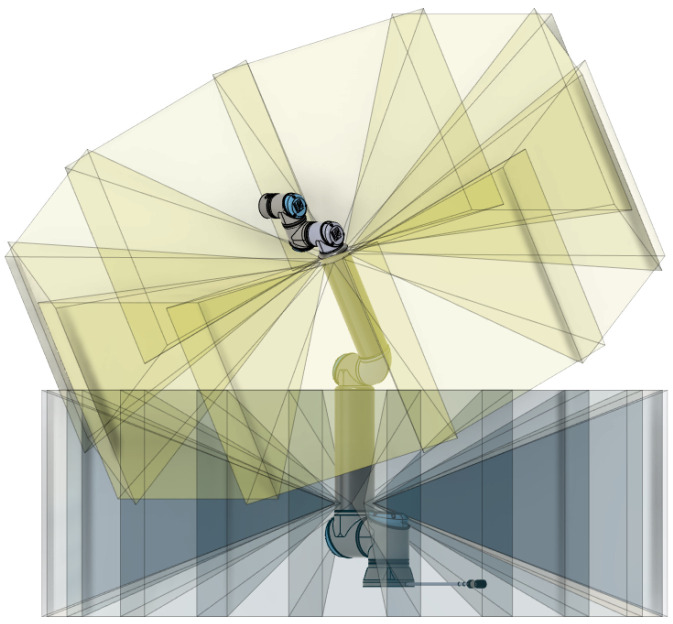
Excerpt of the CAD model of a UR10e robot with the projection of the FoV of two arrays of proximity sensors mounted, respectively, on the first link and on the second link. In this robot configuration, it is evident that the intersection of the FoV of ToF sensors belong to different arrays.

ProxySKIN features a large number of tactile and proximity sensing elements that, once integrated into the rigid links of the robotic platform, as shown in Figure 1, would provide all the necessary information about the surrounding environment, enabling safe and efficient robot operation. In [31], a characterization of the capacitive tactile elements used in our architecture is provided and the measures needed to overcome the electromagnetic interference and the thermal variations affecting the sensors’ readouts are described. In [39], a characterization of a single VL53L8CX ToF is presented, and the maximum ranging capabilities of the sensor under different ambient light conditions are reported. However, there is no reference to tests with multiple proximity sensors and to the interference problems that might occur when intersecting the Field of Illumination (FoI) of multiple sensing elements. In our application, this test is essential to ensure safe and reliable data acquisition from the proximity sensors for two main reasons:

- The Field of View (FoV) of adjacent ToF sensors in an array is partially overlapped to locally minimize the blind volume not monitored by the proximity sensors, as explained in Section 4.1;
- The FoV of proximity sensors placed on different links of the robot might intersect depending on the joint configuration, as shown in Figure 11.

To this aim, two tests are carried out to assess the performance of the Time-of-Flight sensors embedded in ProxySKIN:

1. Interference test among ToF sensors belonging to the same array;
2. Interference test between distinct arrays of ToF sensors.

These characterization experiments provide important information about the sensors’ behaviour and are essential for the development of our distributed sensing architecture, because they prove that several proximity sensors can be integrated into a common, unified framework and can be effectively used to enhance robot performance and cognitive capabilities.

### 5.1. Interference Test among ToF Sensors Belonging to the Same Array

**Figure 12:**
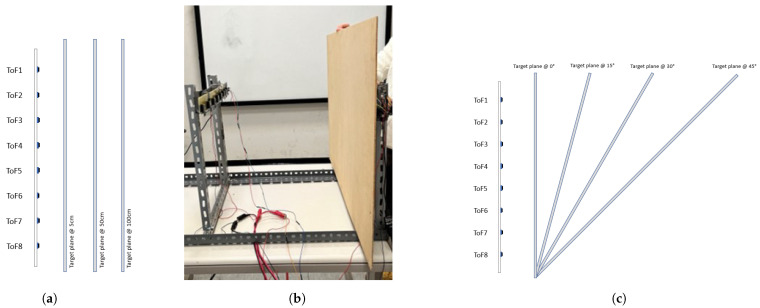
Interference tests among proximity sensors belonging to the same array. (**a**) *Test 1*: The target plane, parallel with respect to the linear array of ToF sensors, is placed at various distances. (**b**) Picture of the real setup. (**c**) *Test 2*: The target plane is set up at different angles with respect to the linear array of proximity sensors.

In this characterization experiment, our aim is to assess the response of a single array of eight proximity sensors, acquiring 500 samples (~35 s) at 15 Hz in the 8 × 8 configuration. The main objective is to check the presence of interference in the measurements of a ToF due to the activity of other proximity sensors belonging to the same array. The tests consider a linear array of ToFs and a target plane placed at different distances (5 cm, 50 cm and 100 cm) and angles (0°, 15°, 30° and 45°) with respect to the sensing devices, as depicted in Figure 12. The tests are performed in three different operational conditions: (a) by connecting only one proximity sensor (ToF3); (b) by enabling four alternated proximity sensors, namely ToF1, ToF3, ToF5, ToF7; (c) and by acquiring data from all eight proximity sensors.

**Figure 13:**
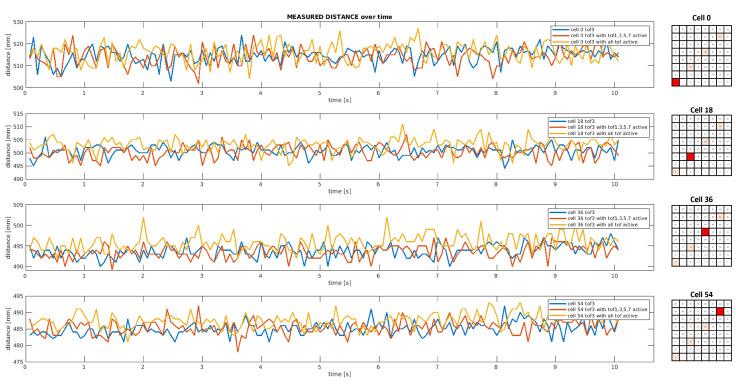
*Test 1*: Plot of 150 distance measurements as a function of time acquired by ToF3 in different operational conditions, considering a parallel target plane placed at 50 cm with respect to the linear array of ToF sensors.

Figure 13 shows some distance measurements collected over time by the third proximity sensor of the linear array considering a parallel target plane placed at 50 cm in three different operating conditions. As highlighted in red on the mesh grids on the right, the plots report just four measurements out of the 64 ranges provided by a single sensor, namely cell 0, cell 18, cell 36 and cell 54. By comparing data collected by ToF3 in the absence of other proximity sensors (blue graph) or in the presence of other sensing devices (orange and yellow graphs), it is clear that the ToF operation is not affected by the activity of other proximity sensors. The interference generated by overlapping the Field of Illumination (FoI) of adjacent ToFs is negligibile.

Table 2 presents a statistical analysis of 500 samples collected by ToF3 for all the tests carried out with the target plane at 0°. Only four measurements are reported for the sake of clarity in two operational conditions and, by comparing them, the results suggest that the distance readouts are not corrupted by light interference due to the activity of other ToF sensors.

**Figure 14:**
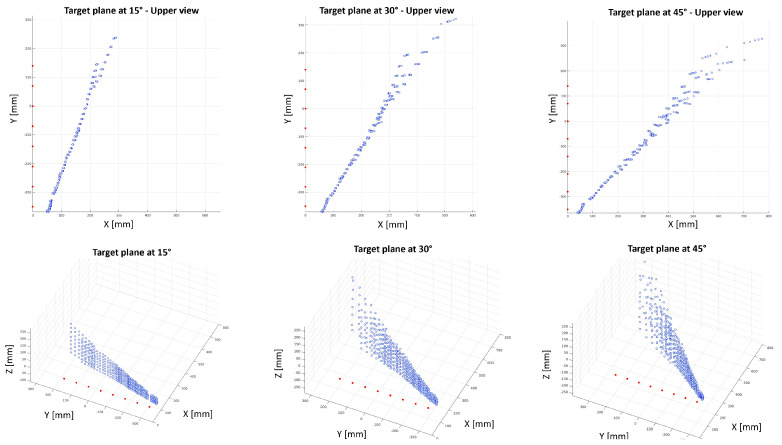
*Test 2*: The 2D and 3D plots of the reconstructed point cloud acquired by eight ToF sensors with target plane set up at different angles. ToF sensor origins are represented with red dots. ToF measurements are represented by blue dots.

Figure 14 shows the 2D and 3D reconstruction of the distance measurements collected by the linear array of eight proximity sensors with a target plane placed at 15°, 30° and 45° with respect to the sensing devices. ToF sensors are depicted as red dots, while the blue point cloud represents a sample of data acquired at a certain instant by all ToF sensors. By analysing the 2D and 3D plots, there is no noticeable interference affecting the sensors measurements.

### 5.2. Interference Test between Distinct Arrays of ToF Sensors

The main goal of this experiment is to assess the response of two different arrays of eight proximity sensors and, in particular, to check the presence of interference in the measurements of a ToF due to the activity of other ToFs. The linear arrays of ToF sensors are placed one in front of the other at different distances, namely 50 cm, 100 cm and 150 cm. Proximity sensors work at 15 Hz in the 8 × 8 configuration and 500 samples (~35 s) are acquired for each test. Tests are performed in two operational conditions: (a) by enabling only one array of ToFs; (b) by simultaneously acquiring data from two arrays of eight ToF sensors.

**Figure 15:**
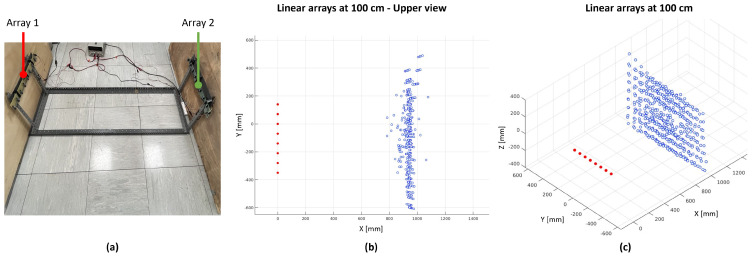
Interference tests between proximity sensors belonging to the different arrays. (**a**) Picture of the real setup. (**b**,**c**) The 2D and 3D reconstructions of the distance measurements collected by eight ToF sensors of array 1, with the data acquisition from array 2 simultaneously enabled.

Figure 15 shows the real setup with two arrays of ToF sensors (a), and the 3D reconstruction of the distance measurements collected by the array on the left when the array on the right is simultaneously enabled (b,c). From a qualitative analysis of the plots, there is no noticeable light interference affecting the distance measurements.

**Figure 16:**
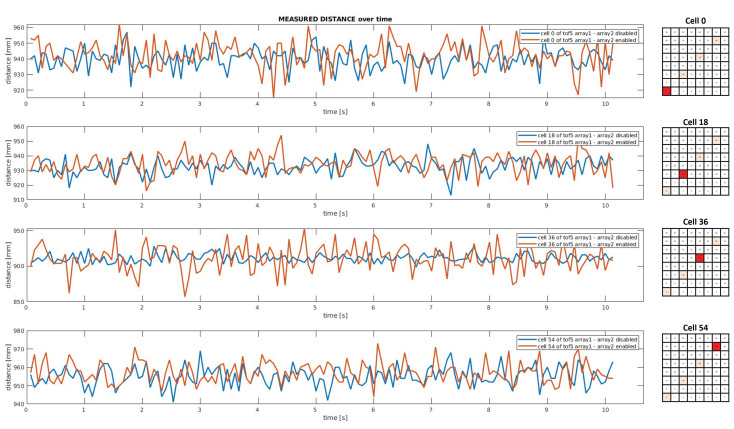
Plot of 150 distance measurements as a function of time acquired by ToF5 of the first array in two different operational conditions: the second array is disabled (blue graph) and enabled (red graph).

In Figure 16, some measurements collected over time by the fifth proximity sensor of one of the linear arrays in two operating conditions are plotted. By comparing data collected by ToF5 when only one array of proximity sensors is enabled (blue line) and when both arrays are simultaneously activated (red line), the results suggest that the distance readouts are not corrupted by light interference due to the activity of other ToF sensors.

## 6. Conclusions

In this paper, we presented the early developments of ProxySKIN, a multi-modal distributed sensing architecture built on top of a well-established tactile technology. We summarized the structure of the CySkin architecture and provided examples of its successful adoption in robotic applications, highlighting its limitations and emphasizing the need for a proper multi-modal evolution. We thoroughly described the preliminary analysis that guided the design towards a ring-shaped embedded structure realized with a flexible PCB hosting 10 ToF sensors. Technical details on the physical realization of the devices and the communication architecture handling both tactile and proximity sensors were presented. In the end, we provided a preliminary experimental evaluation of how Field of Illumination (FoI) interference affects the proximity measurements, a fundamental aspect towards the realization of a multi-sensor array. The results showed that when all the proximity sensors are enabled, the mutual effects of interference are negligible. In particular, distance measurements are affected at the scale of millimeters, which is perfectly acceptable in a typical human–robot interaction application. The next steps in our research will include a complete characterization of the integrated proximity rings, comprehensive sensitivity to different light conditions, electrical noise and power consumption. Moreover, we will integrate several patches of ProxySKIN onto a collaborative robot and evaluate the robustness of the EtherCAT architecture in different network configurations, also considering a single communication backbone for both tactile and proximity sensors. ProxySKIN will then be tested and validated on three relevant use-case scenarios—specifically in automotive, logistics and agricultural applications—aligned with the objectives of the SestoSenso project.

## Figures and Tables

**Table 1:** Analysis of the blind volume $V_{tot}\;\left[m^{3}\right]$ not covered by the ToF sensors, as a function of the number of ToFs per array and the number of arrays, considering a reference volume $V_{ref}=3.746\;m^{3}$.

		# arrays						
		1 array	2 arrays		3 arrays		4 arrays	
			*aligned*	*misaligned*	*aligned*	*misaligned*	*aligned*	*misaligned*
**# ToFs**	8 ToFs	2.421	1.456	1.417	1.379	1.231	1.365	1.159
	10 ToFs	2.253	1.162	1.173	1.067	1.067	1.049	1.047
	12 ToFs	2.240	1.143	1.142	1.044	1.044	1.024	1.024
	14 ToFs	2.234	1.135	1.135	1.036	1.036	1.015	1.015
	16 ToFs	2.230	1.130	1.130	1.032	1.032	1.010	1.010

**Table 2:** Statistical analysis of 500 distance measurements acquired by ToF3 in different tests and operational conditions. Mean values (μ) and standard deviations (σ) are expressed in millimeters [mm].

		cell 0		cell 18		cell 36		cell 54	
		μ[mm]	σ[mm]	μ[mm]	σ[mm]	μ[mm]	σ[mm]	μ[mm]	σ[mm]
50 mm	Config 1 ToF3 enabled	55.583	1.300	51.565	1.1555	54.360	1.273	53.866	1.442
	Config 2 all ToFs enabled	55.583	1.299	51.565	1.155	54.358	1.272	53.866	1.442
500 mm	Config 1 ToF3 enabled	514.252	23.42	500.40	22.59	493.40	22.23	485.01	21.90
	Config 2 all ToFs enabled	515.89	23.58	509.85	23.25	505.84	23.01	487.09	22.04
1000 mm	Config 1 ToF3 enabled	988.70	47.24	1010.63	45.82	995.92	44.88	980.68	44.42
	Config 2 all ToFs enabled	994.30	48.88	1012.78	46.11	999.11	45.09	982.95	44.65

## Data Availability

Data are contained within the article.
